# Supplementary material for: Structural dynamics of human fatty acid synthase in the condensing cycle
Source: Nature. 2025 Feb 20;641(8062):529–36. doi: 10.1038/s41586-025-08782-w (PMC12058526; doi:10.1038/s41586-025-08782-w)
Supplement: Supplementary file 1 — Supplementary Figs. 1–9. [file 41586_2025_8782_MOESM1_ESM.pdf]

nature portfolio

https://doi.org/10.1038/s41586-025-08782-w

Supplementary information

Structural dynamics of human fatty acid synthase in the condensing cycle

In the format provided by the authors and unedited

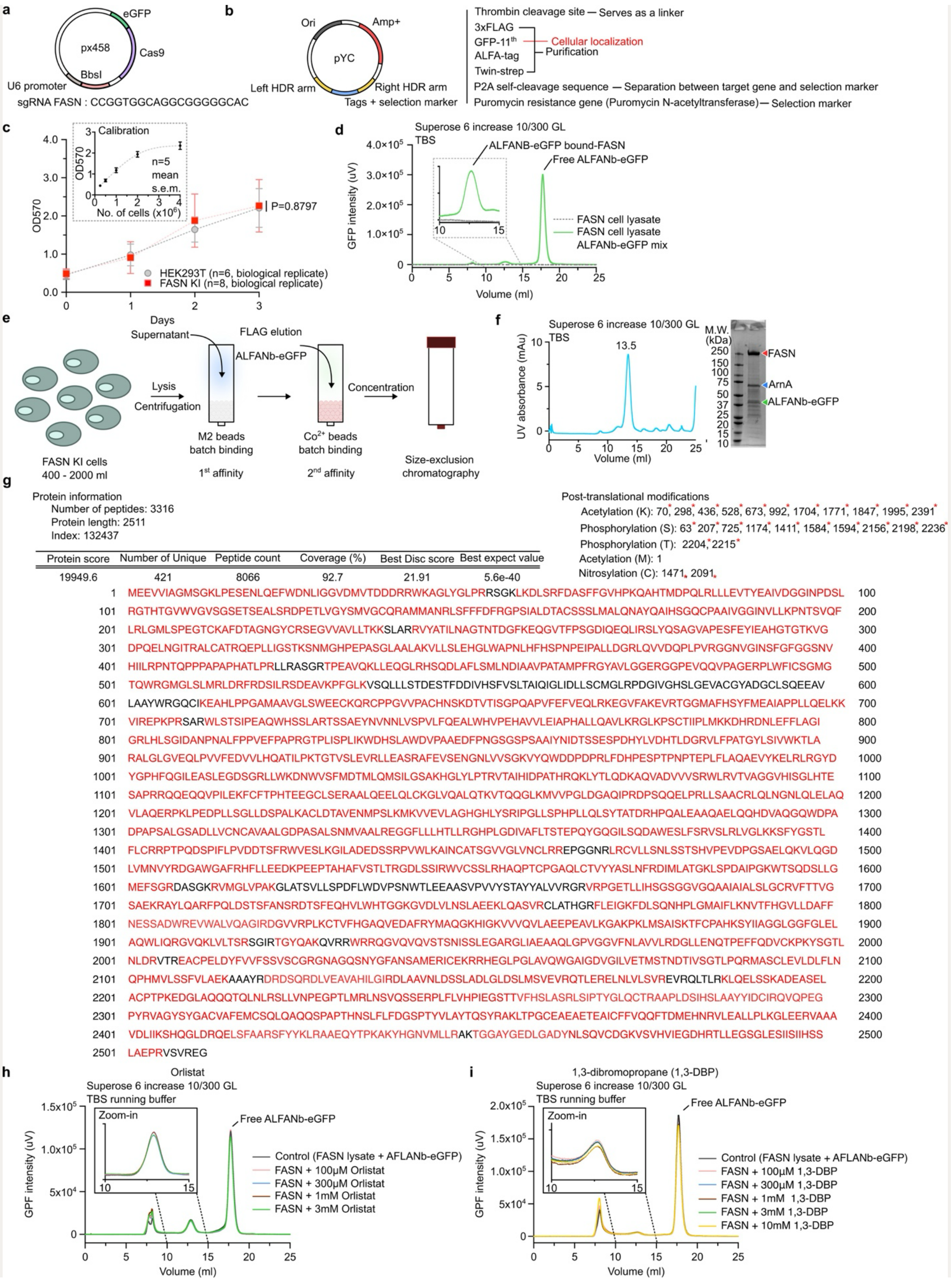
a
eGFP
px458
Cas9
BbsI
U6 promoter
sgRNA FASN : CCGGTGGCAGGCGGGGGCAC
b
Ori
Amp+
pYC
Left HDR arm
Right HDR arm
Tags + selection marker
Thrombin cleavage site — Serves as a linker
3xFLAG
GFP-11th
Cellular localization
ALFA-tag
Purification
Twin-strep
P2A self-cleavage sequence — Separation between target gene and selection marker
Puromycin resistance gene (Puromycin N-acetyltransferase)— Selection marker
c
Calibration
OD570
n=5
mean
s.e.m.
No. of cells (x10^6)
P=0.8797
HEK293T (n=6, biological replicate)
FASN KI (n=8, biological replicate)
Days
d
Superose 6 increase 10/300 GL
TBS
ALFANB-eGFP bound-FASN
Free ALFANb-eGFP
GFP intensity (uV)
FASN cell lyasate
FASN cell lyasate
ALFANb-eGFP mix
Volume (ml)
e
Supernatant
FLAG elution
ALFANb-eGFP
Lysis
Centrifugation
Concentration
M2 beads
batch binding
1st affinity
Co2+ beads
batch binding
2nd affinity
FASN KI cells
400 - 2000 ml
Size-exclusion
chromatography
f
Superose 6 increase 10/300 GL
TBS
13.5
UV absorbance (mAu)
Volume (ml)
M.W.
(kDa)
FASN
ArnA
ALFANb-eGFP
g
Protein information
Number of peptides: 3316
Protein length: 2511
Index: 132437
Protein score | Number of Unique | Peptide count | Coverage (%) | Best Disc score | Best expect value
19949.6 | 421 | 8066 | 92.7 | 21.91 | 5.6e-40
Post-translational modifications
Acetylation (K): 70,* 298,* 436,* 528,* 673,* 992,* 1704,* 1771,* 1847,* 1995,* 2391*
Phosphorylation (S): 63* 207,* 725,* 1174,* 1411,* 1584,* 1594,* 2156,* 2198,* 2236*
Phosphorylation (T): 2204,* 2215*
Acetylation (M): 1
Nitrosylation (C): 1471,* 2091*
1 MEEVVIAGMSGKLPESENLQEFWDNLIGGVDMVTDDDRRWKAGLYGLPRRSGKLKDLSRFDASFFGVHPKQAHTMDPQLRLLLEVTYEAIVDGGINPDSL 100
101 RGTHTGVWVGVSGSETSEALSRDPETLVGYSMVGCQRAMMANRLSFFFDFRGPSIALDTACSSSLMALQNAYQAIHSGQCPAAIVGGINVLLKPNTSVQF 200
201 LRLGMLSPEGTCKAFDTAGNGYCRSEGVVAVLLTKKSLARRVYATILNAGTNTDGFKEQGVTFPSGDIQEQLIRSLYQSAGVAPESFEYIEAHGTGTKVG 300
301 DPQELNGITRALCATRQEPLLIGSTKSNMGHPEPASGLAALAKVLLSLEHGLWAPNLHFHSPNPEIPALLDGRLQVVDQPLPVRGGNVGINSFGFGGSNV 400
401 HIILRPNTQPPPAPAPHATLPRLLRASGRTPEAVQKLLEQGLRHSQDLAFLSMLNDIAAVPATAMPFRGYAVLGGERGGPEVQQVPAGERPLWFICSGMG 500
501 TQWRGMGLSLMRLDRFRDSILRSDEAVKPFGLKVSQLLLSTDESTFDDIVHSFVSLTAIQIGLIDLLSCMGLRPDGIVGHSLGEVACGYADGCLSQEEAV 600
601 LAAYWRGQCIKEAHLPPGAMAAVGLSWEECKQRCPPGVVPACHNSKDTVTISGPQAPVFEFVEQLRKEGVFAKEVRTGGMAFHSYFMEAIAPPLLQELKK 700
701 VIREPKPRSARWLSTSIPEAQWHSSLARTSSAEYNVNNLVSPVLFQEALWHVPEHAVVLEIAPHALLQAVLKRGLKPSCTIIPLMKKDHRDNLEFFLAGI 800
801 GRLHLSGIDANPNALFPPVEFPAPRGTPLISPLIKWDHSLAWDVPAAEDFPNGSGSPSAAIYNIDTSSESPDHYLVDHTLDGRVLFPATGYLSIVWKTLA 900
901 RALGLGVEQLPVVFEDVVLHQATILPKTGTVSLEVRLLEASRAFEVSENGNLVVSGKVYQWDDPDPRLFDHPESPTPNPTEPLFLAQAEVYKELRLRGYD 1000
1001 YGPHFQGILEASLEGDSGRLLWKDNWVSFMDTMLQMSILGSAKHGLYLPTRVTAIHIDPATHRQKLYTLQDKAQVADVVVSRWLRVTVAGGVHISGLHTE 1100
1101 SAPRRQQEQQVPILEKFCFTPHTEEGCLSERAALQEELQLCKGLVQALQTKVTQQGLKMVVPGLDGAQIPRDPSQQELPRLLSAACRLQLNGNLQLELAQ 1200
1201 VLAQERPKLPEDPLLSGLLDSPALKACLDTAVENMPSLKMKVVEVLAGHGHLYSRIPGLLSPHPLLQLSYTATDRHPQALEAAQAELQQHDVAQGQWDPA 1300
1301 DPAPSALGSADLLVCNCAVAALGDPASALSNMVAALREGGFLLLHTLLRGHPLGDIVAFLTSTEPQYGQGILSQDAWESLFSRVSLRLVGLKKSFYGSTL 1400
1401 FLCRRPTPQDSPIFLPVDDTSFRWVESLKGILADEDSSRPVWLKAINCATSGVVGLVNCLRREPGGNRLRCVLLSNLSSTSHVPEVDPGSAELQKVLQGD 1500
1501 LVMNVYRDGAWGAFRHFLLEEDKPEEPTAHAFVSTLTRGDLSSIRWVCSSLRHAQPTCPGAQLCTVYYASLNFRDIMLATGKLSPDAIPGKWTSQDSLLG 1600
1601 MEFSGRDASGKRVMGLVPAKGLATSVLLSPDFLWDVPSNWTLEEAASVPVVYSTAYYALVVRGRVRPGETLLIHSGSGGVGQAAIAIALSLGCRVFTTVG 1700
1701 SAEKRAYLQARFPQLDSTSFANSRDTSFEQHVLWHTGGKGVDLVLNSLAEEKLQASVRCLATHGRFLEIGKFDLSQNHPLGMAIFLKNVTFHGVLLDAFF 1800
1801 NESSADWREVWALVQAGIRDGVVRPLKCTVFHGAQVEDAFRYMAQGKHIGKVVVQVLAEEPEAVLKGAKPKLMSAISKTFCPAHKSYIIAGGLGGFGLEL 1900
1901 AQWLIQRGVQKLVLTSRSGIRTGYQAKQVRRWRRQGVQVQVSTSNISSLEGARGLIAEAAQLGPVGGVFNLAVVLRDGLLENQTPEFFQDVCKPKYSGTL 2000
2001 NLDRVTREACPELDYFVVFSSVSCGRGNAGQSNYGFANSAMERICEKRRHEGLPGLAVQWGAIGDVGILVETMSTNDTIVSGTLPQRMASCLEVLDLFLN 2100
2101 QPHMVLSSFVLAEKAAAYRDRDSQRDLVEAVAHILGIRDLAAVNLDSSLADLGLDSLMSVEVRQTLERELNLVLSVREVRQLTLRKLQELSSKADEASEL 2200
2201 ACPTPKEDGLAQQQTQLNLRSLLVNPEGPTLMRLNSVQSSERPLFLVHPIEGSTTVFHSLASRLSIPTYGLQCTRAAPLDSIHSLAAYYIDCIRQVQPEG 2300
2301 PYRVAGYSYGACVAFEMCSQLQAQQSPAPTHNSLFLFDGSPTYVLAYTQSYRAKLTPGCEAEAETEAICFFVQQFTDMEHNRVLEALLPLKGLEERVAAA 2400
2401 VDLIIKSHQGLDRQELSFAARSFYYKLRAAEQYTPKAKYHGNVMLLRAKTGGAYGEDLGADYNLSQVCDGKVSVHVIEGDHRTLLEGSGLESIISIIHSS 2500
2501 LAEPRVSVREG
h
Orlistat
Superose 6 increase 10/300 GL
TBS running buffer
Zoom-in
Free ALFANb-eGFP
GPF intensity (uV)
Control (FASN lysate + AFLANb-eGFP)
FASN + 100μM Orlistat
FASN + 300μM Orlistat
FASN + 1mM Orlistat
FASN + 3mM Orlistat
Volume (ml)
i
1,3-dibromopropane (1,3-DBP)
Superose 6 increase 10/300 GL
TBS running buffer
Zoom-in
Free ALFANb-eGFP
GPF intensity (uV)
Control (FASN lysate + AFLANb-eGFP)
FASN + 100μM 1,3-DBP
FASN + 300μM 1,3-DBP
FASN + 1mM 1,3-DBP
FASN + 3mM 1,3-DBP
FASN + 10mM 1,3-DBP
Volume (ml)

Supplementary Figure. 1 | Endogenous FASN tagging, purification and verification.
a and b, Plasmids design of px458 and pYC for generating Cas9 RNP complex with corresponding sgRNA (**a**) and homology directed repair donor (**b**). **c**, Upper: calibration curve was obtained from five biological replicates. The center values are the mean and the error bar is standard error of the mean (s.e.m.) as labeled in the corresponding figure. Bottom: validation of FASN stable cell line with endogenous FASN tagged at its C-terminus. Growth rate of wildtype HEK293 cells (gray) and FASN-tagged cells (red). The number of biological replicates to wildtype HEK and FASN-tagged HEK is 6 and 8, respectively. Unpaired parametric Two-tail student t-test is implemented to identify P-value, as labeled in the figure. The center values are the mean and the error bar is standard deviation. Prism is used to generate the graph and calculate statistics. **d**, Fluorescence-detection size-exclusion chromatography (FSEC) of cell lysate. FSEC was enabled by binding ALFA nanobody conjugated with GFP. Endogenous FASN is detected as dimer. The experiment is repeated five times. **e**, Protein purification scheme. **f**, SEC and corresponding SDS-PAGE. Human endogenous FASN is marked. Major contaminants are identified (Supplementary Fig. 3) and marked. Ten times of independent protein purifications have been performed. **g**, LC-MS validation of purified endogenous FASN with 92.7 % sequence coverage (red). Post-translational modifications (PTM) identified by LC-MS are listed. Residues labeled in red asterisk contains previous reported PTM. **h** and **i**, FSEC profile of cell lysate with different concentrations of Orlistat and 1,3-DBP. The experiment is repeated three times and Prism is used to generate the graphs in this figure.

.

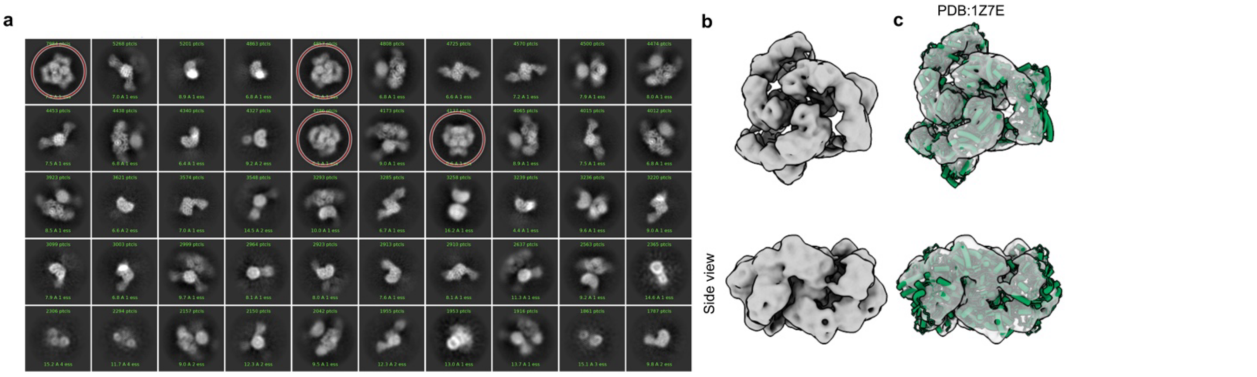

d

Rank	ACC#	Protein Score	Peptide Count	Number Unique	% Cov	Best Disc Score	Best Expected Val	Protein Lenght	Protein MW	Species	Protein Name
5	P77398	1684.6	38	101	52.9	8.71	1.6e-15	660	74289.5	ECOLI	Bifunctional polymyxin resistance protein ArnA

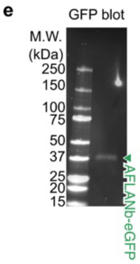

Supplementary Figure. 2 | Contaminants in purified endogenous FASN.

a, 2D class averages from the dataset collected from the purified FASN, shown in Supplementary Figure. 1f. None FASN particles are marked by red circle. **b**, Two different views of the reconstruction determined from non-FASN particles in the dataset. **c**, Same two views of the density map in transparent with the crystal structure of bacterial ArnA (PDB:1Z7E) docked. **d**, LC-MS identifies the contaminant as bacterial ArnA. e, The 37kDa band is confirmed as ALFANb-eGFP contaminant using GFP blotting (Excitation 488 nm).

a

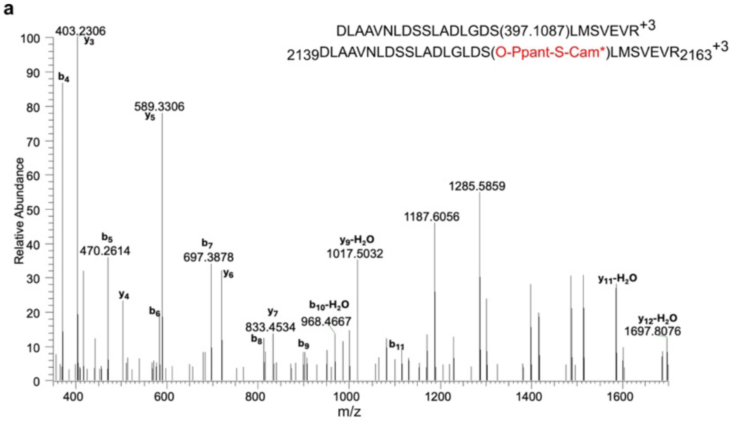

b

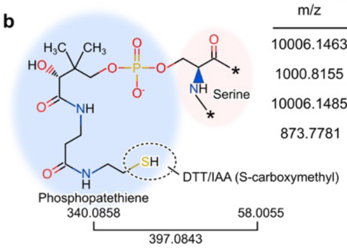

m/z	z	ppm	Peptide	Start	End	Score	Expect	Modification
10006.1463	3	0	DLAAVNLDSSLADLGLDS(397.1062)LM(Oxidation)SVEVR	2139	2163	34.8	1.5e-6	Ppant
1000.8155	3	0	DLAAVNLDSSLADLGLDS(397.1087)LMSVEVR	2139	2163	36.0	5.0e-7	Ppant
10006.1485	3	0	DLAAVNLDSSLADLGLDS(397.1128)LM(Oxidation)SVEVR	2139	2163	29.3	7.1e-6	Ppant
873.7781	3	0.60	DLAAVNLDSSLADLGLDSLM(Oxidation)SVEVR	2139	2163	32.2	2.9e-8	Intact

c

m/z	z	ppm	Peptide	Start	End	Score	Ppant (340.0858)
997.8026	3	0	DLAAVNLDSSLADLGLDS(388.0700)LMSVEVR	2139	2163	19.2	+ Acetyl moiety (C_2H_3O) 388.0858
1008.8119	3	0	DLAAVNLDSSLADLGLDS(405.1030)LM(Oxidation)SVEVR	2139	2163	32.7	
1007.1425	3	0	DLAAVNLDSSLADLGLDS(416.0897)LMSVEVR	2139	2163	31.2	
1014.4731	3	0	DLAAVNLDSSLADLGLDS(422.0866)LM(Oxidation)SVEVR	2139	2163	22.2	+ Malonyl moiety (C_3HO_3) 425.0858
1022.8209	3	0	DLAAVNLDSSLADLGLDS(447.1300)LM(Oxidation)SVEVR	2139	2163	27.7	
773.6300	4	0	DLAAVNLDSSLADLGLDS(472.1800)LM(Oxidation)SVEVR	2139	2163	32.2	
1034.1696	3	0	DLAAVNLDSSLADLGLDS(481.1761)LM(Oxidation)SVEVR	2139	2163	22.9	Intermediate products
1039.1682	3	0	DLAAVNLDSSLADLGLDS(496.1719)LM(Oxidation)SVEVR	2139	2163	39.3	
859.1660	4	0	DLAAVNLDSSLADLGLDS(814.3240)LM(Oxidation)SVEVR	2139	2163	17.2	
1150.2155	3	0	DLAAVNLDSSLADLGLDS(829.3138)LM(Oxidation)SVEVR	2139	2163	29.2	
894.4381	4	0	DLAAVNLDSSLADLGLDS(971.4071)LM(Oxidation)SVEVR	2139	2163	51.7	

Increasing molecular mass on Ppant-modified S2156

Supplementary Figure. 3 Validation of Phosphopantetheine modification on 2156Serine.

a, Raw data of LC-MS peaks on Phosphopantetheine (Ppant)-modified S2156. **b**, Ppant is labeled with S-carboxymethyl (left) to enable identification of Ppant modification in S2156 (right). Purified endogenous FASN contains both apo- and holo-ACP. **c**, List of peptides with increased molecular weights correspond to acyl chain of different sizes attached to the tip of Ppant.

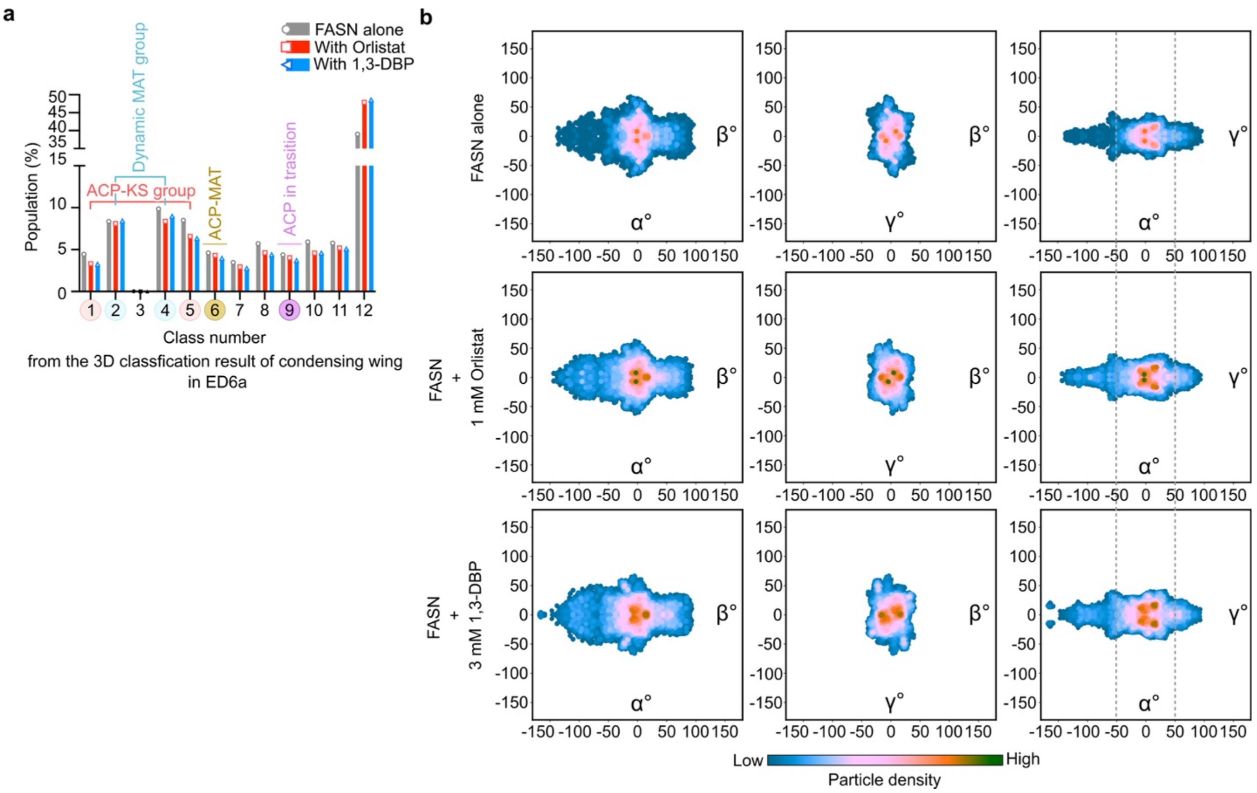

Supplementary Figure. 4 | Classification data on condensing domain, and conformational landscape of three dataset. a, Populations of all classes identified during symmetry expanded classification (Extended Data Fig. 6a) in three different datasets, FASN alone (gray), with Orlistat (red) and 1,3-DBP (blue). Prism is used to generate the graph. **b,** conformational landscape of three different datasets. Axes of each panel are labeled with degree (°) as the unit.

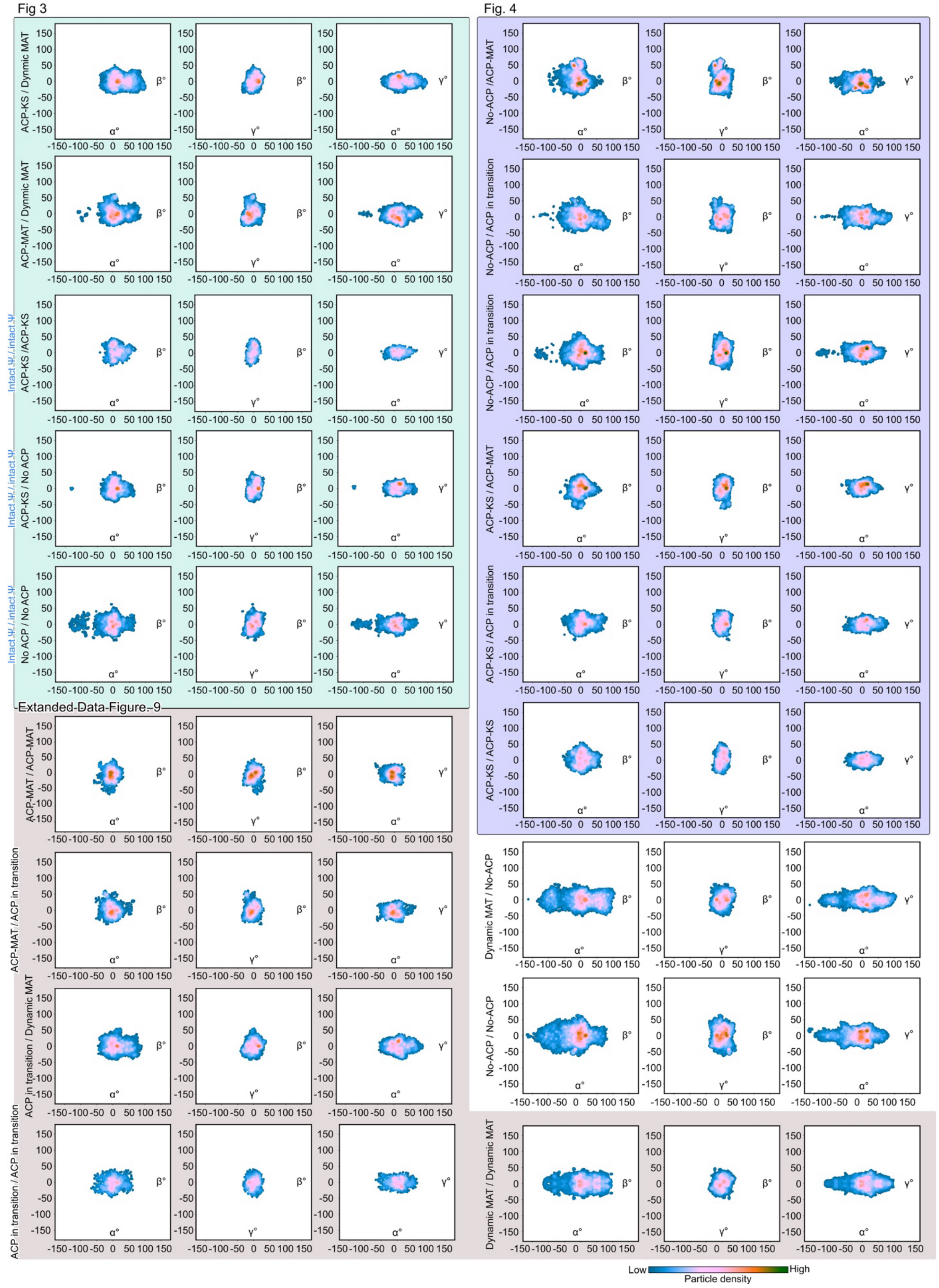

Supplementary Figure. 5 | Full-view of conformational landscape used in this study.

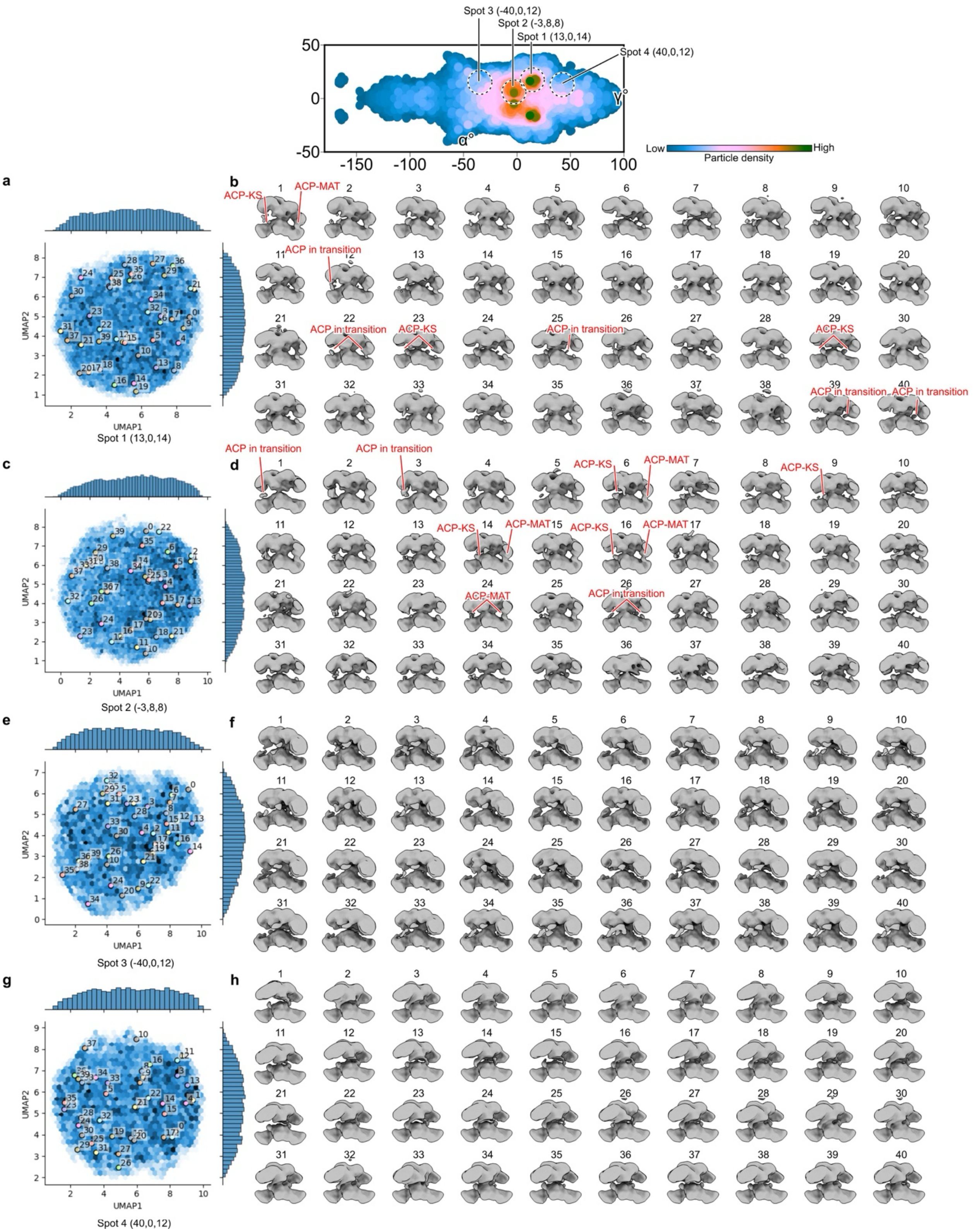

Supplementary Figure. 6 | CryoDRGN analysis.

Real space overall conformational landscape of all particles. Particles from four different spots in the landscape are selected for cryoDRGN analysis, spot 1 for **a**, 2 for **c**, 3 for **e** and 4 for **g**. **a, c, e**

and g, UMAP visualization of the latent distribution of all particles with a defined orientation between the condensing and modifying domain after training of an 8-dimensional latent variable model with cryoDRGN. Dots present latent space coordinates, from which reconstruction was calculated. **b**, **d**, **f** and **h**, Maps calculated from the designated position in the corresponding UMAP. Axes of each panel are labeled with degree (°) as the unit.

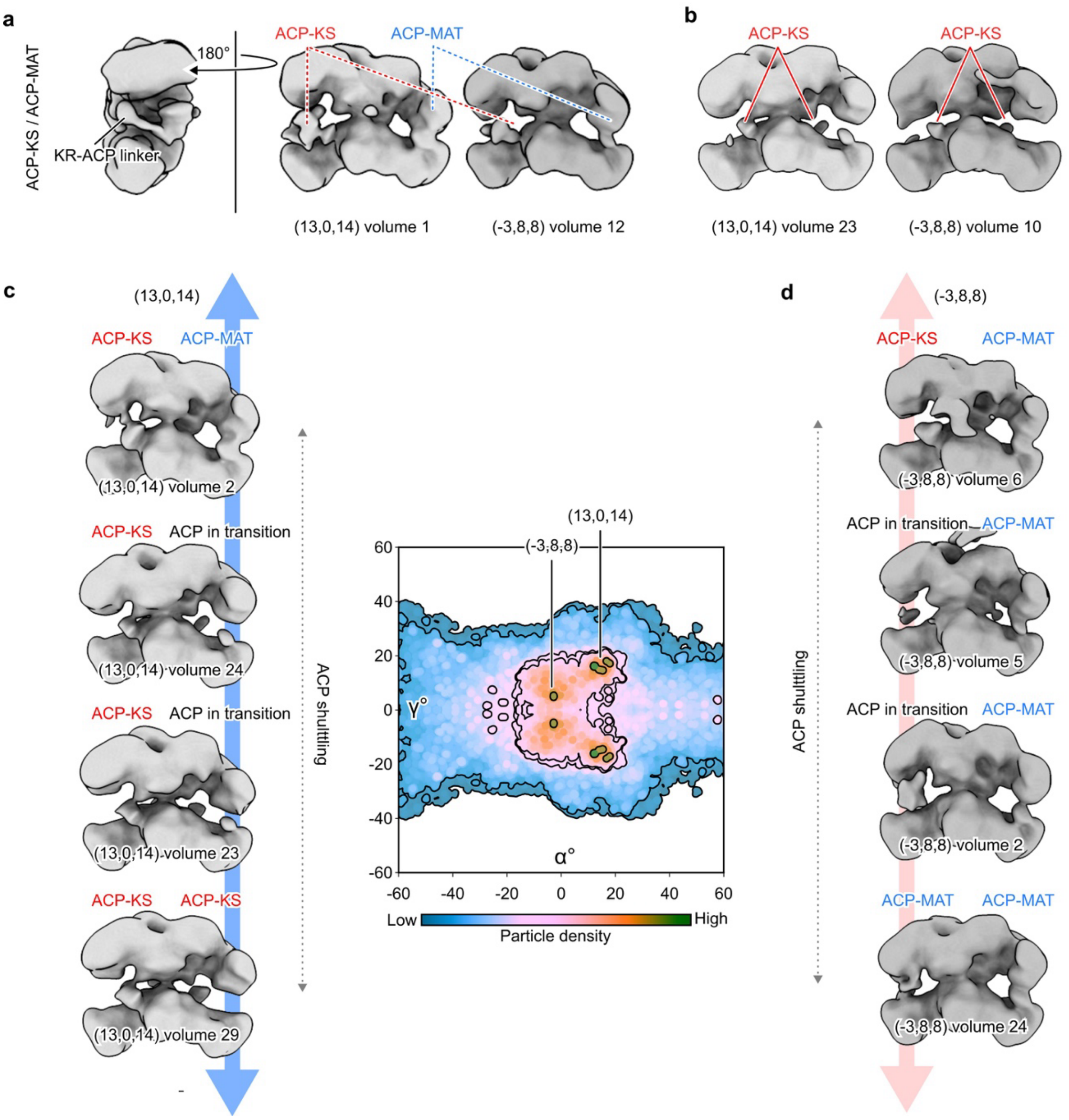

Supplementary Figure. 7 | Structural flexible analysis using cryoDRGN.
a, Reconstructions selected from classes calculated from cryoDRGN analysis of particles around the coordinate (13°, 0°, 14°) in the conformational landscape of the combined dataset that includes all substates and all three datasets. **b**, Reconstructions selected from classes calculated from cryoDRGN analysis of particles around another coordinate (-3°, 8°, 8°) in the conformational landscape of the combined dataset that includes all substates and all three datasets. **c and d**, In both cases, ACP seem being shuttled without large rotational motion. A display of the complete classes is in the **Supplementary Fig. 6**. Axes of each panel are labeled with degree (°) as the unit.

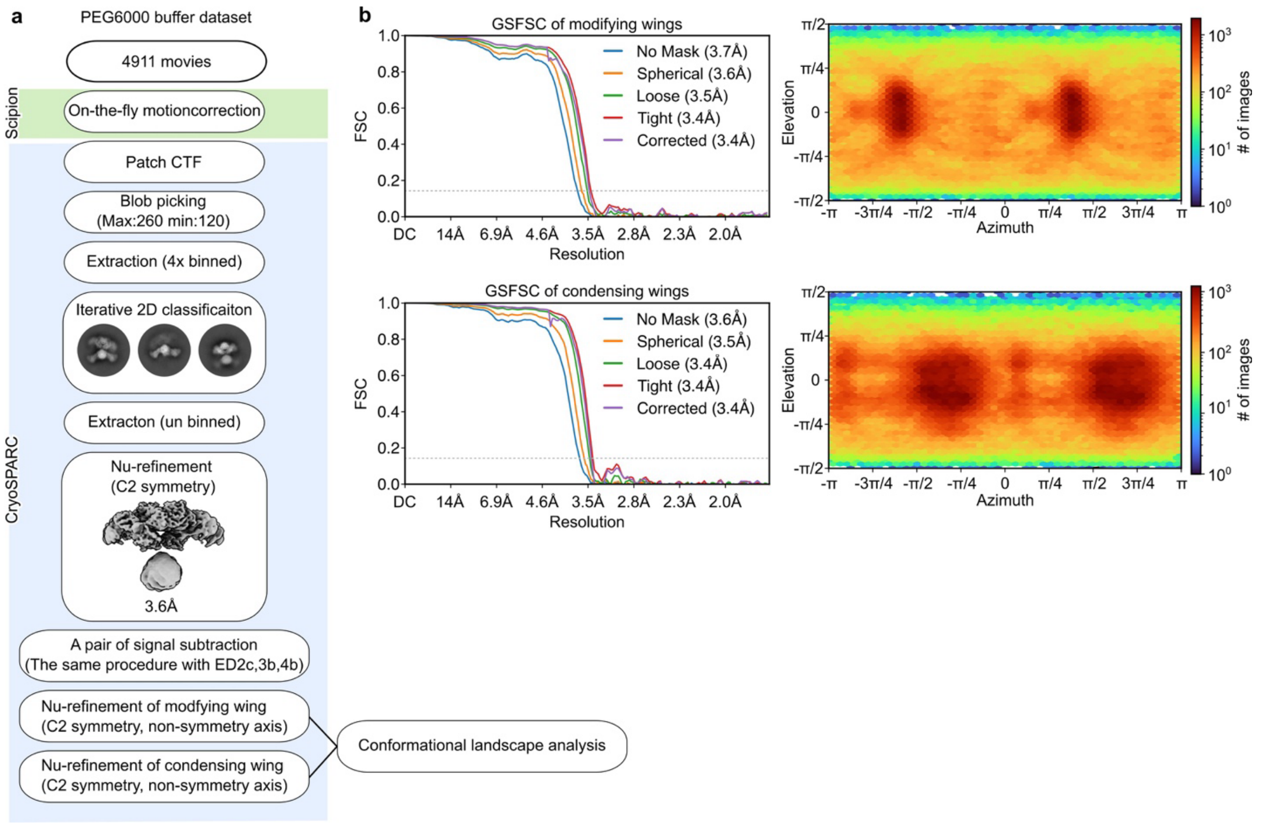

Supplementary Figure. 8 | Data processing workflow of endogenous FASN in PEG6000 buffer.

a, Single particle cryo-EM data processing workflow of endogenous FASN in PEG6000 buffer. **b,** FSC curves of modifying and condensing wings (left) and angular distribution of particles for each reconstruction (right).

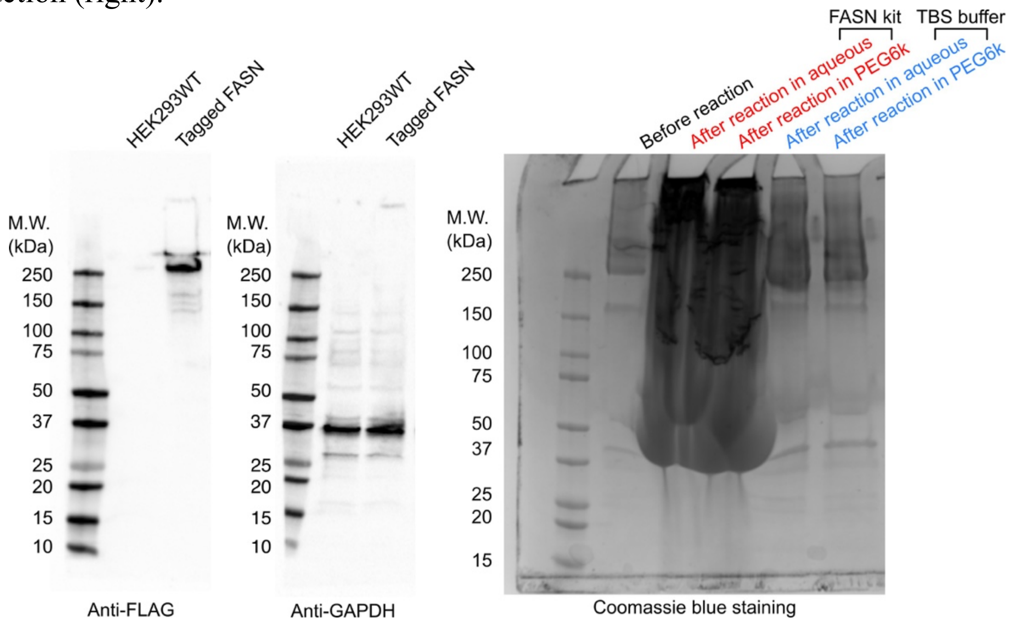

Supplementary Figure. 9 | Raw image of SDS-PAGEs in Fig. 1c and Extended Data Figure 1d.

Supplementary Video 1 | Docked Rat ACP into ACP-KS group. Rat ACP (PDB: 2PNG) is docked into the ACP density in ACP-KS group. Two different positions of ACP, position 1 (salmon) and position 2 (grey) are displayed using the same view with **Fig 2d**. Rat ACP (white cylinder helix bundle) is docked into the ACP density. Position 1 (salmon) shows a density inserted into the catalytic cavity, marked as Ppant. In position 2 (grey), a helical density from ACP engages with H68 and K70 of KS. The same color code is used with **Fig 2d**. The movie is generated to use ChimeraX.

Supplementary Video 2 | Docked Rat ACP into ACP-MAT group. Rat ACP (PDB: 2PNG) is docked into the ACP density in ACP-MAT group. Movie displays ACP-MAT engagement using the same view with **Fig 2g.** The same color code is used with **Fig 2g.** The movie is generated to use ChimeraX.

Supplementary Video 3 | Docked Rat ACP into ACP in transition. Rat ACP (PDB: 2PNG) is docked into the ACP density in ACP in transition group. Movie displays ACP in transition using the same view with **Fig 2h.** The same color code is used with **Fig 2h.** The movie is generated to use ChimeraX.
